# Oxidative Stress and PRKN-Mediated Senescence Link RhoA/ROCK Signaling to Epithelial Remodeling in Allergic Rhinitis

**DOI:** 10.3390/antiox15010077

**Published:** 2026-01-07

**Authors:** Xuan Yuan, Wei Zhong, Shaobing Xie, Liyuan Liu, Wenjing Gu, Yixiang Zeng, Hua Zhang, Weihong Jiang, Zhihai Xie, Peisong Gao

**Affiliations:** 1 Division of Allergy and Clinical Immunology, Johns Hopkins University School of Medicine, Baltimore, MD 21224, USA; xyuan30@jh.edu (X.Y.); shaobing@csu.edu.cn (S.X.); lliu138@alumni.jh.edu (L.L.); wgu16@jh.edu (W.G.); yzeng36@jh.edu (Y.Z.); 2 Department of Otolaryngology Head and Neck Surgery, Xiangya Hospital of Central South University, 87 Xiangya Road, Changsha 410008, China; 258102175@csu.edu.cn (W.Z.); entxy@126.com (H.Z.); jiangwh68@126.com (W.J.); 3 Department of Respiratory Medicine, Children’s Hospital of Soochow University, Suzhou 215000, China; 4 Hunan Province Key Laboratory of Otolaryngology Critical Diseases, Xiangya Hospital of Central South University, Changsha 410008, China

**Keywords:** allergic rhinitis, ROS, RhoA/ROCK, mitochondrial function, cellular senescence, epithelial remodeling, PRKN

## Abstract

Allergic rhinitis (AR) is characterized by persistent epithelial remodeling, yet the upstream drivers and molecular mechanisms remain poorly defined. Analysis of nasal mucosa from AR patients revealed marked epithelial remodeling, oxidative stress, and Th2 inflammation. Transcriptome analysis of nasal mucosa revealed RhoA as one of the most upregulated genes, with expression positively correlating with disease severity. Using epithelial-specific RhoA-deficient mice (*RhoA^cKO^*) and fasudil, a RhoA/ROCK inhibitor, we found that loss of RhoA/ROCK signaling markedly attenuated nasal Th2 inflammation, oxidative stress, and epithelial remodeling following allergen challenge. Further transcriptome analysis demonstrated that elevated RhoA activation was associated with increased epithelial cellular senescence. Both in vitro and in vivo studies confirmed that epithelial RhoA activation promotes allergen- or Th2 cytokine-induced cellular senescence, whereas genetic or pharmacologic elimination of senescent cells alleviated allergic inflammation and tissue remodeling. Pathway analysis identified PRKN (parkin) as a central node within RhoA-regulated, senescence-associated networks in AR. Functional studies showed that PRKN overexpression mitigated IL-13-induced mitochondrial dysfunction, oxidative stress, and epithelial senescence in human nasal epithelial cells. Together, these findings reveal that RhoA-driven epithelial senescence contributes to allergic inflammation and epithelial remodeling in AR and identify PRKN as a potential therapeutic target to restore epithelial homeostasis.

## 1. Introduction

Allergic rhinitis (AR) is a chronic inflammatory disorder of the upper airway affecting over 500 million individuals worldwide [1,2]. While AR is primarily driven by type 2 immune responses, characterized by elevated interleukin (IL)-4, IL-5, and IL-13, accumulating evidence suggests that structural alterations in the nasal epithelium, such as epithelial thickening, barrier dysfunction, and goblet cell metaplasia, play a pivotal role in disease persistence, symptom severity, and susceptibility to environmental triggers [3,4,5]. However, the cellular and molecular mechanisms underlying epithelial remodeling in AR remain incompletely defined.

The small GTPase Ras homolog family member A (RhoA) is a key intracellular signal transducer belonging to the Rho family of small GTPases [6]. It functions as a nucleotide-dependent molecular switch, cycling between an inactive GDP-bound state and an active GTP-bound state [7]. Upon activation, RhoA interacts with various downstream effectors, such as Rho-associated coiled-coil containing protein kinase (ROCK), to regulate actomyosin contractility, junctional integrity, and epithelial barrier function [8,9,10]. Notably, activation of the RhoA/ROCK pathway has been implicated in airway inflammation by modulating the recruitment, differentiation, and activation of multiple inflammatory cell types, including eosinophils, macrophages, mast cells, and T cells [11,12]. Recent studies further indicate that dysregulated RhoA/ROCK signaling contributes to epithelial barrier disruption, airway inflammation, and tissue remodeling in diverse inflammatory airway diseases [8,9,13]. However, the specific role of epithelial RhoA signaling in AR, particularly its contribution to epithelial remodeling through non-immune mechanisms, remains largely unexplored.

Beyond its immunomodulatory functions, emerging evidence indicates that RhoA signaling is a key regulator of oxidative stress and mitochondrial homeostasis—processes intimately linked to epithelial cellular senescence [8,14]. Epithelial senescence has recently been recognized as a hallmark of chronic airway inflammation [15,16,17]. Senescent epithelial cells exhibit impaired proliferative capacity and secrete a broad array of pro-inflammatory cytokines, chemokines, and matrix-degrading enzymes, collectively termed the senescence-associated secretory phenotype (SASP) [17,18,19]. Excessive accumulation of reactive oxygen species (ROS) is a well-established trigger of cellular senescence and epithelial dysfunction [20,21]. While these mechanisms have been implicated in diseases such as asthma, pulmonary fibrosis, and chronic obstructive pulmonary disease (COPD) [21,22,23], the presence and functional significance of nasal epithelial senescence in AR remain largely undefined. Moreover, the contribution of epithelial RhoA/ROCK signaling to the induction of senescence and epithelial remodeling in AR has not yet been elucidated.

In this study, we sought to define how RhoA/ROCK signaling contributes to epithelial oxidative stress, senescence, and remodeling in AR, and to identify the molecular pathways underlying these processes. Nasal mucosa from AR patients was examined histologically and by bulk RNA sequencing to identify RhoA-associated transcriptomic changes. Epithelial-specific *RhoA*-deficient mice and the ROCK inhibitor fasudil were used to evaluate the effects of RhoA/ROCK inhibition on oxidative stress, cellular senescence, and epithelial remodeling. The contribution of senescent cells to allergic inflammation, oxidative stress, and epithelial remodeling was also investigated. Pathway and network analyses identified *PRKN*, a key regulator of mitochondrial homeostasis [24], as a major downstream mediator of RhoA signaling. Functional assays in human nasal epithelial cells (HNEpCs) further validated the role of *PRKN* in modulating mitochondrial dysfunction, oxidative stress, and epithelial senescence.

## 2. Materials and Methods

### 2.1. Human Study Subjects

This study enrolled 40 participants, including 20 patients with AR and 20 healthy controls (HCs). The diagnosis of AR was established according to the 2023 International Consensus Statement on Allergy and Rhinology: Allergic Rhinitis (ICAR-Allergic Rhinitis 2023) criteria [25]. All AR patients were confirmed to be sensitized to house dust mites (HDMs) by skin prick testing. HCs were non-atopic individuals who underwent nasal surgery for non-inflammatory, non-allergic conditions. Exclusion criteria included prior allergen immunotherapy, concurrent infection, pregnancy, or use of systemic corticosteroids, antihistamines, or immunosuppressive agents within four weeks before sample collection. Inferior turbinate mucosal biopsies were obtained intraoperatively. Mucus sampling was performed by placing a sterile 1 × 2 cm Leukosorb filter paper (Pall Scientific, Port Washington, NY, USA) between the middle turbinate and nasal septum under endoscopic guidance, left in place for 3 min. Papers were then centrifuged at 4 °C for 30 min, and mucus from both sides was pooled and stored at −80 °C [26,27]. Symptom severity in AR patients was evaluated using the Total Nasal Symptom Score (TNSS) and the Rhinoconjunctivitis Quality of Life Questionnaire (RQLQ) [28,29,30]. Clinical and demographic characteristics of all participants are summarized in Table S1 in the Online Repository. The study protocol was approved by the Human Research Ethics Committee of Xiangya Hospital, Central South University, and written informed consent was obtained from all participants prior to enrollment.

### 2.2. Mice

Wild-type (WT) C57BL/6J mice, *CC10-CreER^TM^* transgenic mice, and *p16-3MR* homozygous mice were obtained from The Jackson Laboratory (Bar Harbor, ME, USA). *RhoA^f^*^/^*^f^* mice were generously provided by Dr. Yi Zheng (University of Cincinnati). To generate conditional RhoA knockout mice, *CC10-CreER^TM^* mice were crossed with *RhoA^f^*^/^*^f^* mice to obtain *CC10-CreER^TM^*; *RhoA^f^*^/^*^f^* (*RhoA^cKO^*) mice. Offspring were genotyped by PCR using primers specific for the *Cre* transgene and floxed *RhoA* alleles to identify *RhoA^cKO^* mice. Mice positive for both *Cre* and *flox* alleles were selected for experiments. All mice were maintained under specific pathogen-free conditions at the animal facility of the Johns Hopkins University School of Medicine. All experimental procedures were conducted in accordance with the guidelines of the National Institutes of Health and were approved by the Institutional Animal Care and Use Committee of Johns Hopkins University.

### 2.3. Generation of the AR Murine Model

The murine model of AR was established following a modified protocol from previous studies [31,32]. Both *RhoA^cKO^* and *Rho ^flf^* mice received tamoxifen (75 mg/kg; Sigma-Aldrich, St. Louis, MO, USA) once daily for five consecutive days, followed by a 7-day washout period to induce Cre-mediated gene recombination in airway club cells. Mice were then sensitized by intraperitoneal injection of 100 μg HDM (Greer Laboratories, Lenoir, NC, USA) emulsified in 2 mg Imject Alum (Thermo Fisher Scientific, Waltham, MA, USA) in 200 μL PBS on days 0, 7, and 14. From days 21 to 26, mice were challenged intranasally with 50 μg HDM in 20 μL PBS once daily for six consecutive days, while controls received PBS alone. *p16-3MR* mice underwent the same protocol and were treated with ganciclovir (GCV; 25 mg/kg; MedChemExpress, Monmouth Junction, NJ, USA) or vehicle (DMSO) once daily during the challenge phase to selectively eliminate senescent cells. In a separate experiment, AR mice received the ROCK inhibitor fasudil (30 mg/kg; MedChemExpress) 30 min before each HDM challenge [9,10]. Mice were euthanized on day 27, and nasal tissues were collected for histological and molecular analyses. Nasal lavage fluid (NALF) was collected by gently perfusing the nasal cavity with 1 mL of cold PBS via the trans-pharyngeal route; the recovered fluid was centrifuged at 700× *g* for 10 min at 4 °C, and the supernatant was stored at −80 °C [32,33]. Serum and NALF were used to quantify total and HDM-specific IgE and cytokine levels. Allergic symptoms were behaviorally assessed by counting sneezing and nasal scratching events, with no baseline behavioral differences observed between *RhoA^f^*^/^*^f^* and *RhoA^cKO^* mice, within 10 min after the final intranasal challenge.

### 2.4. Hematoxylin and Eosin and Periodic Acid–Schiff Staining

Fresh human inferior turbinate tissues and mouse nasal specimens were fixed in 4% paraformaldehyde, dehydrated, paraffin-embedded, and sectioned at 4 μm thickness. Tissue sections were dewaxed, rehydrated, and subjected to Hematoxylin and Eosin (H&E) and Periodic Acid–Schiff (PAS) staining using commercially available kits (Abcam, Cambridge, UK), according to the manufacturer’s instructions [9].

### 2.5. Human Nasal Epithelial Cells Culture and Intervention

Primary human nasal epithelial cells (NECs) were isolated from inferior turbinate tissues obtained from HCs during surgery and cultured in PneumaCul^TM^-Ex Plus Medium (STEMCELL Technologies, Vancouver, BC, Canada) at 37 °C in a humidified 5% CO_2_ incubator, as previously described [34,35]. When cells reached approximately 80% confluence, they were dissociated and seeded into 6-well plates at a density of 1 × 10^5^ cells/well.

### 2.6. Mouse NEC Air–Liquid Interface Culture

Primary mouse NECs were isolated and cultured based on established protocols [34,36]. NECs were initially seeded in Collagen I-coated flasks (Corning, NY, USA) and expanded in PneumaCult^TM^-Ex Plus Medium with daily medium replacement. Upon reaching 80% confluence, NECs were dissociated and seeded at a density of ~1 × 10^5^ cells/well onto 12-well Transwell inserts (0.4 µm pore size; Corning). Once confluent, an air–liquid interface (ALI) was established by replacing the apical medium with air and culturing with PneumaCult™-ALI Medium (STEMCELL Technologies). Cells were maintained for 21 days to allow full differentiation. Transepithelial electrical resistance (TEER) was measured using an epithelial voltohmmeter (Millipore, Billerica, MA, USA) [34,36].

### 2.7. Establishment of PRKN-Overexpressing HNEpCs

HNEpCs were transduced with a lentiviral vector carrying the full-length human PRKN coding sequence (LV-plex-MCS backbone, NM_004562, 1442 bp; OBiO Technology, Shanghai, China) according to the manufacturer’s instructions. Following transduction, cells were cultured in fresh growth medium containing selection antibiotics for 10–14 days, with medium replaced every 2–3 days, until resistant colonies were established. Surviving clones were expanded and screened for stable PRKN overexpression using quantitative reverse transcription PCR (qRT-PCR) and Western blotting. The verified stable cell lines were used for subsequent experiments.

### 2.8. qRT-PCR

Total RNA was extracted from human nasal tissues using TRIzol reagent (Invitrogen, Waltham, MA, USA), and RNA purity and concentration were assessed spectrophotometrically. Complementary DNA was synthesized via reverse transcription, followed by amplification using the TaqMan^®^ Gene Expression Kit (Thermo Fisher Scientific). GAPDH served as internal control. Relative gene expression was quantified using the 2^−ΔΔCT^ method. Primer sequences are listed in Table S2 in the Online Repository.

### 2.9. Western Blot Assay

Total protein was extracted from nasal mucosal tissues or cultured cells, separated by SDS–PAGE (Servicebio, Wuhan, China), and transferred to PVDF membranes. Membranes were blocked and incubated overnight at 4 °C with primary antibodies against RhoA-GTPase (New East Biosciences, Malvern, PA, USA), PRKN (Cell Signaling Technology, Danvers, MA, USA), and β-Tubulin (Affinity, Changzhou, China). After incubation with HRP-conjugated secondary antibodies, bands were visualized using enhanced chemiluminescence and quantified using ImageJ (version 1.54).

### 2.10. ELISA

Concentrations of IL-4, IL-5, IL-13, IL-1β, IL-6, and total IgE were measured using commercial ELISA kits (BD Biosciences, San Diego, CA, USA) following the manufacturer’s instructions. HDM-specific IgE was quantified by coating 96-well plates with HDM extract (10 µg/mL), followed by blocking, incubation with diluted samples, and detection using biotinylated anti-mouse IgE, streptavidin-HRP, and TMB substrate [9,37,38]. Absorbance was measured using a microplate reader (Bio-Rad, Hercules, CA, USA)

### 2.11. Immunohistochemistry and Immunofluorescence Staining

Immunostaining was performed on paraffin-embedded tissue sections and cultured NECs. After deparaffinization and antigen retrieval, samples were blocked and incubated overnight at 4 °C with primary antibodies against human IL-4, IL-5, and IL-13 (1:200, Affinity). For immunohistochemistry, sections were treated with HRP-conjugated secondary antibodies and developed using DAB substrate, followed by hematoxylin counterstaining. For immunofluorescence staining, primary antibodies (summarized in Table S3, Online Repository) were detected using species-specific fluorophore-conjugated secondary antibodies. Nuclei were counterstained with DAPI. Senescent cells were detected using an SA-β-Gal staining kit (Cell Signaling Technology) according to the manufacturer’s instructions. ROS levels in cultured cells were measured using CM-H_2_DCFDA (total intracellular ROS; Thermo Fisher Scientific) and MitoSOX Red (mitochondrial superoxide; Thermo Fisher Scientific). All images were captured using a fluorescence microscope, and quantification of fluorescence intensity and positive cell percentage was conducted using ImageJ software.

### 2.12. Bulk RNA-Seq Analysis

Total RNA was extracted from nasal mucosal tissues using TRIzol reagent (Invitrogen, Waltham, MA, USA), and RNA integrity was assessed with an Agilent 2200 TapeStation (Agilent Technologies, Santa Clara, CA, USA). Library construction and high-throughput sequencing were performed on an Illumina HiSeq 3000 platform (Illumina, San Diego, CA, USA) by RiboBio (Guangzhou, China). Raw reads were processed and aligned to the reference genome, and differentially expressed genes (DEGs) were identified using the DESeq2 package (v1.40.2) in R (v4.3.0). Genes with an adjusted *p*-value < 0.05 and log2(fold change) > log2(1.5) were considered statistically significant. Visualization of transcriptomic data, including heatmaps and volcano plots, was performed in R. Gene Ontology (GO) enrichment analysis was conducted using the clusterProfiler package (v4.6.0) in R. DEGs were grouped based on RhoA transcript levels in AR (high vs. low RhoA expression; *n* = 4 per group), and significantly enriched biological processes were identified using Benjamini–Hochberg-adjusted *p*-values (FDR < 0.05) and a minimum gene count threshold. Sequencing data have been deposited in the GSA-Human database at CNCB (BioProject ID: PRJCA038464).

### 2.13. Transmission Electron Microscope Examination

For ultrastructural analysis, treated HNEpCs were fixed in 2.5% glutaraldehyde overnight at 4 °C, post-fixed in 1% osmium tetroxide for 2 h, and dehydrated through a graded ethanol series. Specimens were embedded in Spurr’s epoxy resin, sectioned, and stained with uranyl acetate and lead citrate. Samples were imaged using a transmission electron microscope (TEM; Hitachi, Tokyo, Japan).

### 2.14. JC-1 Staining

Treated HNEpCs were incubated with 1 µM JC-1 (Thermo Fisher Scientific) working solution for 20 min at 37 °C in the dark and then observed under a fluorescence microscope. Red JC-1 aggregates represent polarized mitochondria, while green monomers indicate depolarized mitochondria. The red/green fluorescence ratio was used to assess mitochondrial integrity.

### 2.15. Statistical Analysis

All data are expressed as mean ± standard error of the mean (SEM). For comparisons between two groups, either an unpaired two-tailed Student’s *t*-test or the Mann–Whitney U test was used, depending on data distribution. For comparisons involving more than two groups, one-way ANOVA followed by Bonferroni’s multiple comparisons test, or the Kruskal–Wallis test for non-parametric data, was applied as appropriate. Correlations between variables were assessed using Spearman’s rank correlation coefficient. Statistical analyses and graph generation were performed using GraphPad Prism 10.0 (GraphPad Software, La Jolla, CA, USA). A *p* value < 0.05 was considered statistically significant.

## 3. Results

### 3.1. Epithelial Remodeling, Oxidative Stress, and Th2 Inflammation Are Prominent Features in AR Nasal Mucosa

**Figure 1:**
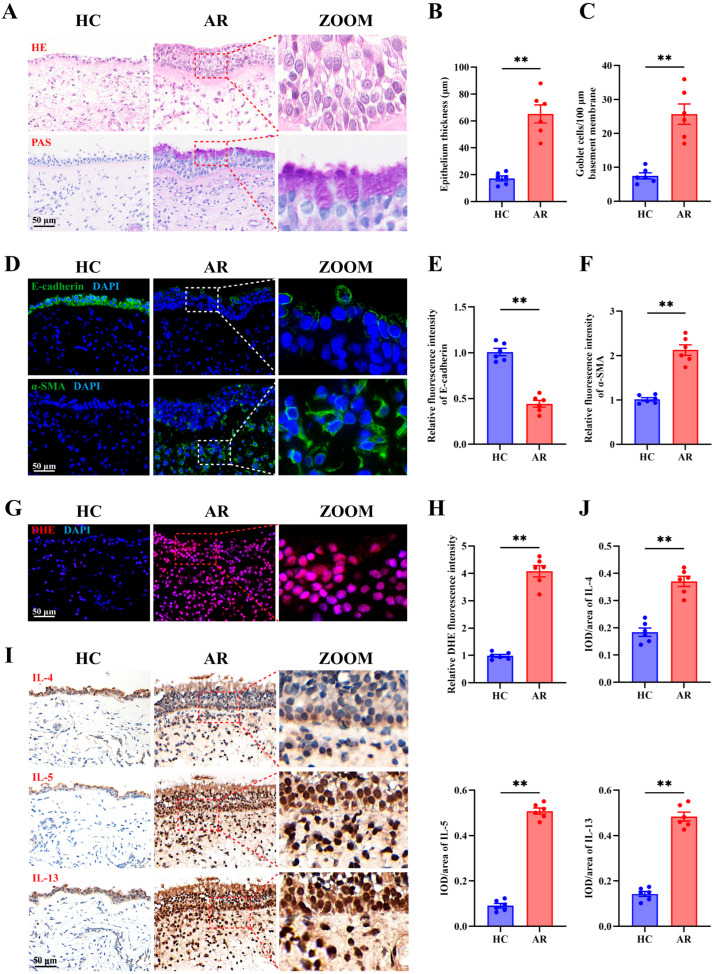
Epithelial remodeling, oxidative stress, and Th2 inflammation are prominent features in AR nasal mucosa. (**A**) Representative images of H&E and PAS staining in HC and AR nasal mucosa. (**B**,**C**) Quantification of epithelial thickness (**B**) and goblet cell density (**C**). (**D**–**F**) Representative images (**D**) and quantification (**E**,**F**) of immunofluorescence staining of E-cadherin and α-SMA expression in HC and AR nasal mucosa. (**G**,**H**) Representative images (**G**) and quantification (**H**) of DHE staining in HC and AR nasal mucosa. (**I**,**J**) Representative images (**I**) and quantification (**J**) of immunohistochemistry staining of IL-4, IL-5, and IL-13 in HC and AR nasal mucosa. *n* = 6. Data are presented as mean ± SEM. ** p < 0.01.

To characterize epithelial alterations and inflammatory features in AR, nasal mucosal biopsies from AR patients and HCs were examined. H&E staining revealed a marked increase in epithelial thickness (Figure 1A,B), and PAS staining showed prominent goblet cell hyperplasia (Figure 1A,C) in the AR group compared with HCs. Immunofluorescence staining demonstrated reduced E-cadherin expression (Figure 1D,E) and elevated α-SMA levels (Figure 1D,F) in AR nasal epithelium, indicative of active epithelial remodeling. ROS accumulation was increased in AR tissues, as shown by enhanced DHE fluorescence (Figure 1G,H). Moreover, immunohistochemical analysis revealed significantly higher expression of Th2-associated cytokines, including IL-4, IL-5, and IL-13, in AR nasal mucosa compared with HCs (Figure 1I,J). Collectively, these findings demonstrate that AR nasal epithelium exhibits marked remodeling, heightened oxidative stress, and a dominant Th2-type inflammatory milieu.

### 3.2. Up-Regulated RhoA in AR Nasal Mucosa and Correlates with Disease Severity

**Figure 2:**
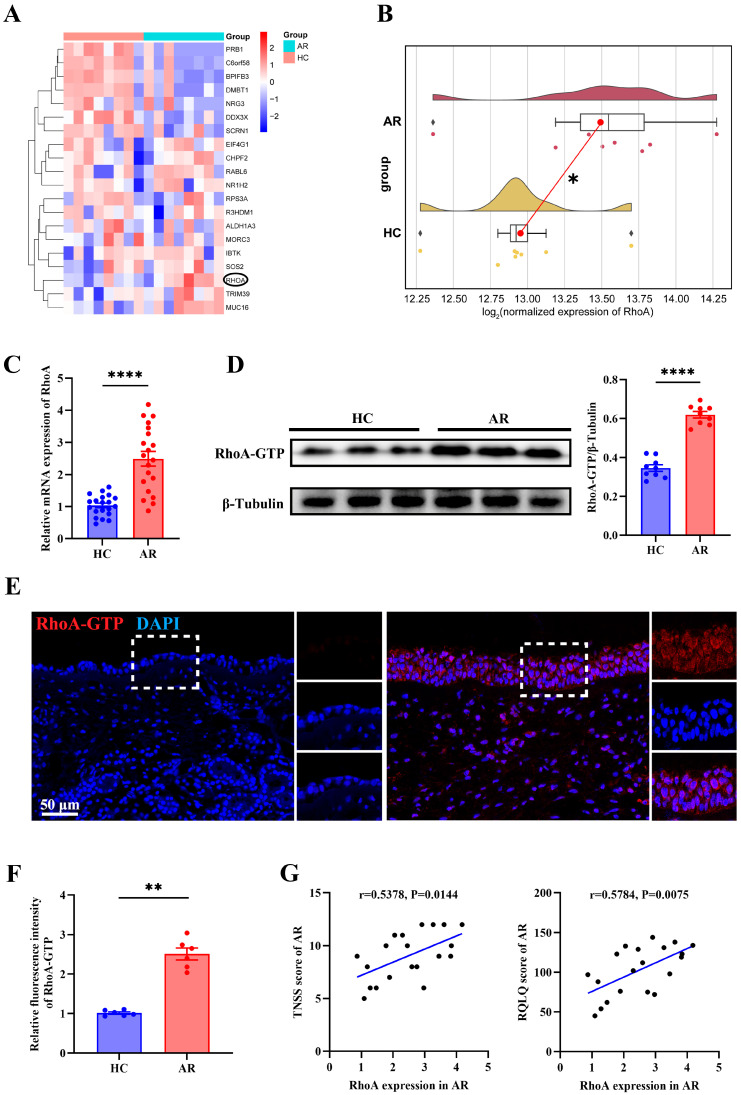
Up-regulated RhoA in AR nasal mucosa and correlates with disease severity. (**A**) Heatmap of differentially expressed genes from nasal mucosa RNA-sequencing of matched AR patients and HCs (*n* = 8). (**B**) Raincloud plots with density overlay of RhoA expression. (**C**) qRT-PCR analysis of RhoA mRNA expression in HC and AR nasal mucosa (*n* = 20). (**D**) Representative Western blot and quantification of RhoA-GTP protein levels in HC and AR groups (*n* = 9). (**E**,**F**) Representative images (**E**) and quantification (**F**) of immunofluorescence staining of RhoA-GTP expression in nasal mucosa (*n* = 6). (**G**) Correlation analysis between RhoA mRNA levels and TNSS and RQLQ in AR patients (*n* = 20). Data are presented as mean ± SEM. * *p* < 0.05, ** *p* < 0.01; **** *p* < 0.0001.

To elucidate the molecular mechanisms driving epithelial remodeling and oxidative stress in AR, we conducted transcriptomic profiling of nasal mucosal tissues from eight matched pairs of AR patients and HCs. Among the differentially expressed genes, RhoA emerged as one of the most significantly upregulated in AR samples (Figure 2A,B). Validation in an expanded cohort confirmed increased RhoA expression in AR nasal tissues (Figure 2C). Consistently, Western blot analysis demonstrated elevated levels of active RhoA (RhoA-GTP) in AR samples compared with HCs (Figure 2D). Immunofluorescence staining further revealed that RhoA-GTP was predominantly localized within the nasal epithelium and was markedly more abundant in AR tissues (Figure 2E,F). Notably, RhoA mRNA levels positively correlated with both TNSS and RQLQ scores in AR patients (Figure 2G), suggesting that epithelial RhoA activation is associated with disease severity. Collectively, these findings indicate that RhoA is upregulated and activated in the nasal mucosa of AR patients.

### 3.3. RhoA/ROCK Signaling Deficiency Attenuates Nasal Th2 Inflammation, Oxidative Stress, and Epithelial Remodeling in AR

**Figure 3:**
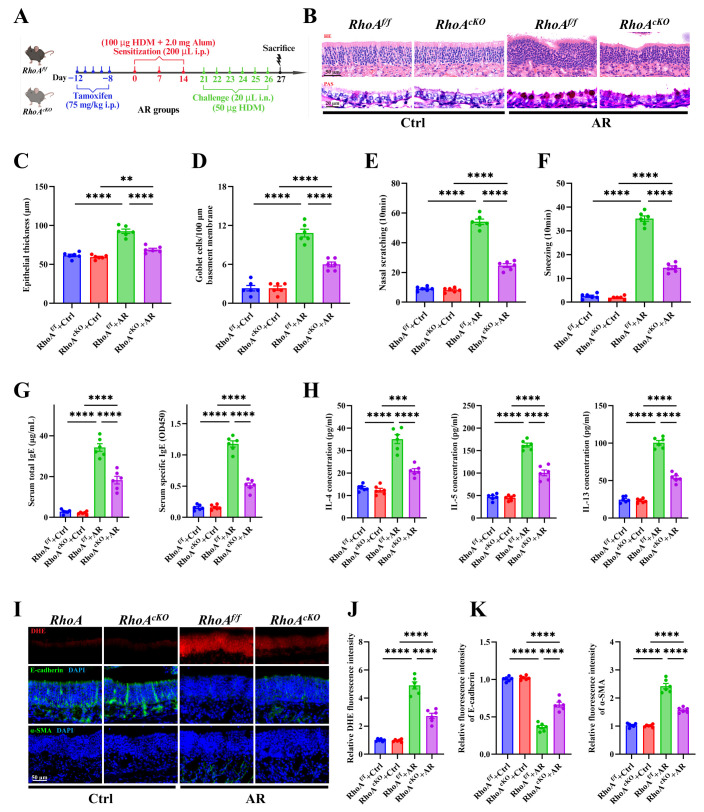
RhoA/ROCK signaling deficiency attenuates nasal Th2 inflammation, oxidative stress, and epithelial remodeling in AR. (**A**) Schematic of HDM-induced AR protocol using tamoxifen-inducible RhoA conditional knockout (*RhoA^cKO^*) and *RhoA^flf^* control mice. (**B**) Representative images of H&E and PAS staining in nasal mucosa. (**C**,**D**) Quantification of epithelial thickness (**C**) and goblet cell density (**D**). (**E**,**F**) Quantification of nasal scratching (**E**) and sneezing frequencies (**F**). (**G**) ELISA quantification of serum total and HDM-specific IgE concentrations. (**H**) ELISA quantification of IL-4, IL-5, and IL-13 levels in nasal lavage fluid. (**I**) Representative images of DHE staining and immunofluorescence staining of E-cadherin and α-SMA expression in nasal mucosa. (**J**,**K**) Quantification of DHE (**J**), E-cadherin and α-SMA (**K**) fluorescence intensity. *n* = 6. Data are presented as mean ± SEM. ** *p* < 0.01, *** *p* < 0.001, **** *p* < 0.0001.

To delineate the functional role of RhoA in epithelial remodeling and Th2 inflammation, we generated tamoxifen-inducible, epithelial-specific RhoA knockout mice (*RhoA^cKO^*) by crossing *RhoA^f^*^/^*^f^* mice with *CC10-CreER^TM^* mice (Figure S1A) [39,40,41]. Successful deletion of RhoA in Club cells was confirmed by genotyping (Figure S1B). Following tamoxifen induction, an HDM-induced AR model was established using these mice (Figure 3A). Compared with control littermates, *RhoA^cKO^* mice exhibited markedly reduced epithelial thickening and goblet cell hyperplasia, as demonstrated by H&E (Figure 3B,C) and PAS staining (Figure 3B,D). Behaviorally, RhoA deficiency significantly decreased nasal scratching (Figure 3E) and sneezing frequencies (Figure 3F). Consistently, serum measurements revealed lower total and HDM-specific IgE levels (Figure 3G), while ELISA analysis of NALF showed reduced IL-4, IL-5, and IL-13 concentrations (Figure 3H). Moreover, *RhoA^cKO^* mice displayed diminished epithelial oxidative stress, evidenced by decreased ROS signals (Figure 3I,J), and mitigated epithelial remodeling, as indicated by preserved E-cadherin and reduced α-SMA expression (Figure 3I,K).

To further validate the role of RhoA/ROCK signaling in AR, we pharmacologically inhibited this pathway using the ROCK inhibitor Fasudil during the challenge phase of the HDM-induced AR model (Figure S2A). Fasudil-treated AR mice recapitulated the protective phenotype observed in *RhoA^cKO^* mice, exhibiting reduced epithelial thickening (Figure S2B,C) and goblet cell metaplasia (Figure S2B,D), along with improved clinical symptoms, including fewer nasal scratching and sneezing events (Figure S2E,F). In parallel, serum total and HDM-specific IgE levels (Figure S2G) and Th2 cytokines (IL-4, IL-5, IL-13) in NALF (Figure S2H) were significantly decreased. Fasudil treatment also reduced ROS accumulation (Figure S2I,J) and restored epithelial remodeling, as indicated by increased E-cadherin and decreased α-SMA expression (Figure S2I,K).

To confirm the epithelial-intrinsic effects of this pathway, primary NECs from WT mice were differentiated under ALI conditions and stimulated with IL-13 (10 ng/mL), with or without Fasudil (10 μM) co-treatment [42,43] (Figure S3A). Fasudil significantly reduced IL-13-induced elevation of intracellular ROS levels, as detected by CM-H_2_DCFDA staining (Figure S3B,C). Immunofluorescence analysis showed that Fasudil reversed IL-13-induced E-cadherin downregulation and α-SMA upregulation (Figure S3B,D,E). Consistently, IL-13 markedly decreased TEER values, reflecting barrier disruption, whereas Fasudil significantly restored TEER values (Figure S3F). Collectively, these results demonstrate that both genetic deletion and pharmacologic inhibition of RhoA/ROCK signaling effectively suppress epithelial remodeling, oxidative stress, and Th2 inflammation in allergic rhinitis.

### 3.4. Elevated RhoA Activation Contributes to Epithelial Senescence in AR Patients

**Figure 4:**
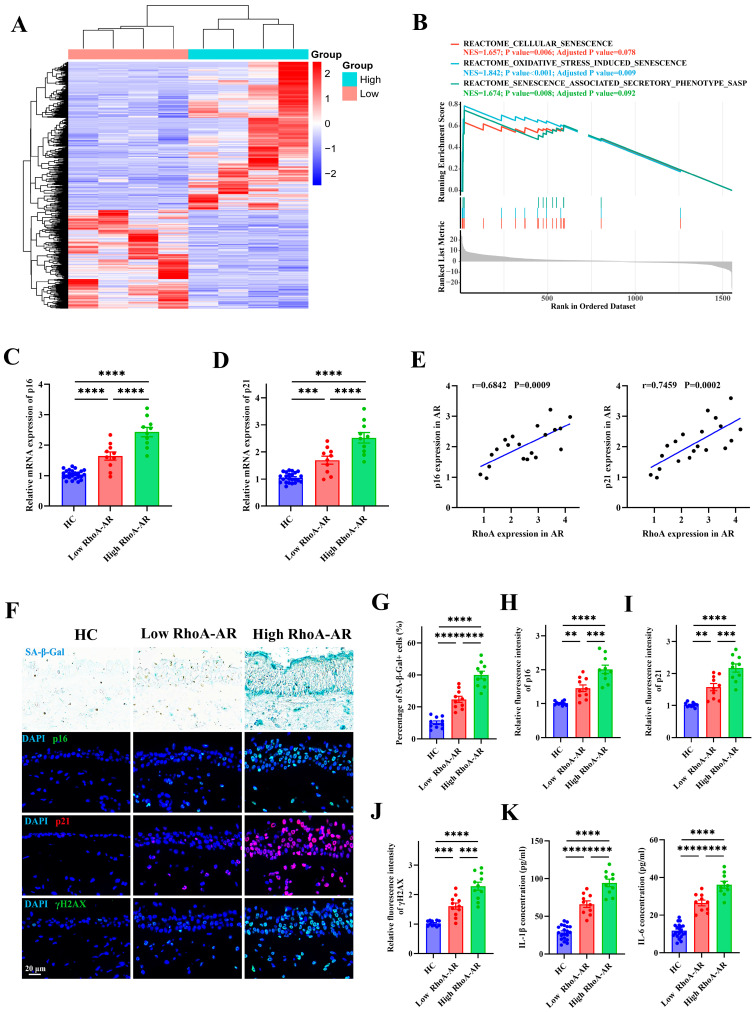
Elevated RhoA activation contributes to epithelial senescence in AR patients. (**A**) Heatmap of differentially expressed genes from RNA-sequencing of AR nasal mucosa grouped by high and low RhoA expression (*n* = 4). (**B**) GSEA enrichment plots of senescence-related pathways in high-RhoA AR samples. (**C**,**D**) qRT-PCR analysis of p16 (**C**) and p21 (**D**) mRNA levels in HC (*n* = 20), low-RhoA AR (*n* = 10), and high-RhoA AR (*n* = 10) nasal mucosa. (**E**) Correlation analysis between RhoA mRNA expression and p16 or p21 levels in AR patients (*n* = 20). (**F**) Representative images of SA-β-Gal staining and immunofluorescence staining of p16, p21, and γH2AX in nasal mucosa from HC, low-RhoA AR, and high-RhoA AR. (**G**–**J**) Quantification of SA-β-Gal-positive cell percentage (**G**) and fluorescence intensity of p16 (**H**), p21 (**I**), and γH2AX (**J**). (**K**) ELISA quantification of IL-1β and IL-6 levels in nasal lavage fluid of AR patients and HCs. Data are presented as mean ± SEM. ** *p* < 0.01; *** *p* < 0.001; **** *p* < 0.0001.

To elucidate the downstream mechanisms by which RhoA contributes to AR pathogenesis, we re-analyzed bulk RNA-sequencing data from AR nasal tissues stratified by RhoA expression levels (high-RhoA vs. low-RhoA, *n* = 4 per group; Figure 4A). Gene Set Enrichment Analysis (GSEA) revealed significant enrichment of senescence-associated pathways, indicating activation of epithelial senescence programs in high-RhoA AR (Figure 4B). Consistently, nasal mucosa from AR patients with high RhoA expression (*n* = 10) exhibited elevated mRNA levels of canonical senescence markers p16 and p21 compared with low-RhoA AR patients (*n* = 10) and HCs (*n* = 20) (Figure 4C,D). Notably, RhoA expression positively correlated with senescence marker levels (Figure 4E). Histochemical SA-β-Gal staining further demonstrated a marked accumulation of senescent cells within the nasal epithelium of AR patients (Figure 4F,G). Immunofluorescence analysis confirmed increased epithelial expression of p16 (Figure 4F,H), p21 (Figure 4F,I), and γH2AX (Figure 4F,J), supporting enhanced epithelial senescence. Moreover, concentrations of the SASP cytokines IL-1β and IL-6 were significantly elevated in NALF from AR patients, with the highest levels observed in those with high RhoA expression (Figure 4K). These results demonstrate that RhoA activation is closely associated with epithelial senescence and SASP induction in AR.

### 3.5. Epithelial RhoA Activation Regulates Allergen or Th2 Cytokine-Induced Cellular Senescence in Both In Vitro and In Vivo Analyses

**Figure 5:**
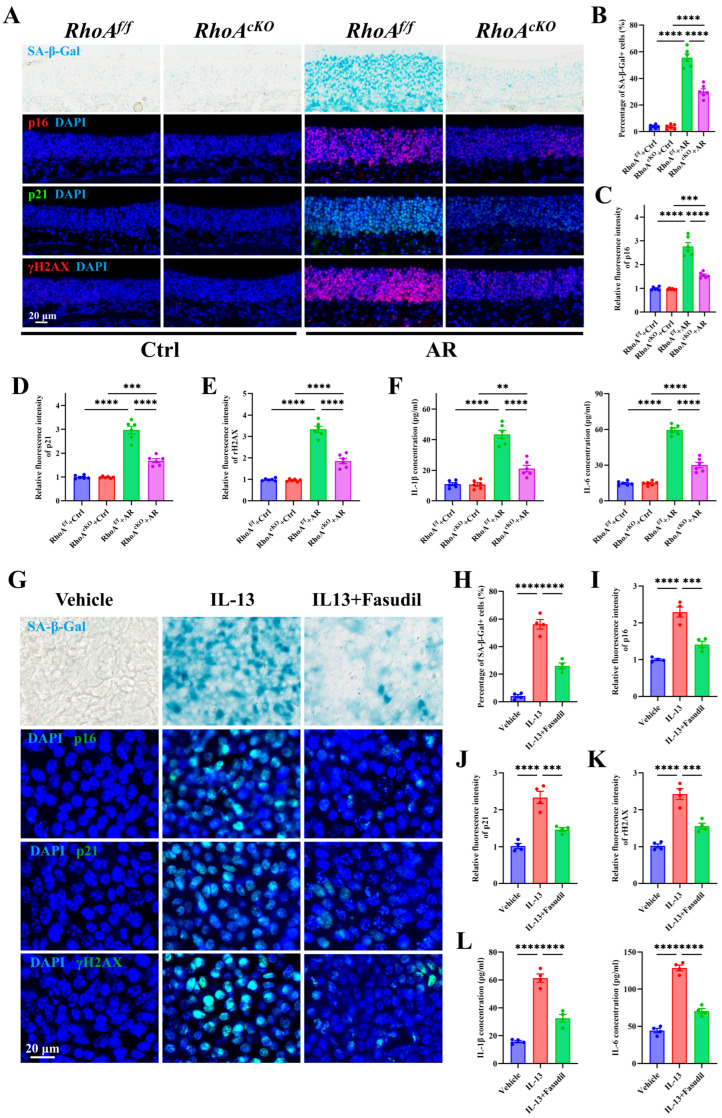
Epithelial RhoA activation regulates allergen or Th2 cytokine-induced cellular senescence in both in vitro and in vivo analyses. (**A**) Representative images of SA-β-Gal staining and immunofluorescence staining of p16, p21, and γH2AX in nasal mucosa. (**B**) Quantification of SA-β-Gal-positive cells in nasal mucosa. (**C**–**E**) Quantification of fluorescence intensity of p16 (**C**), p21 (**D**), and γH2AX (**E**). (**F**) ELISA quantification of IL-1β and IL-6 levels in nasal lavage fluid. n = 6. (**G**) Representative images of SA-β-Gal staining and immunofluorescence staining of p16, p21, and γH2AX in ALI-NECs. (**H**) Quantification of SA-β-Gal-positive cells. (**I**–**K**) Quantification of fluorescence intensity of p16 (**I**), p21 (**J**), and γH2AX (**K**). (**L**) ELISA quantification of IL-1β and IL-6 levels in culture supernatants. *n* = 4. Data are presented as mean ± SEM. ** *p* < 0.01; *** *p* < 0.001; **** *p* < 0.0001.

To determine whether RhoA/ROCK signaling regulates epithelial senescence in AR, we assessed senescence markers in nasal tissues from HDM-induced *RhoA^cKO^* and *RhoA^flf^* mice. SA-β-Gal staining revealed a marked accumulation of senescent epithelial cells in HDM-treated RhoA*^f^*^/^*^f^* mice, which was substantially reduced in *RhoA^cKO^* mice (Figure 5A,B). Similarly, the expression of canonical senescence markers p16 (Figure 5A,C), p21 (Figure 5A,D), and γH2AX (Figure 5A,E) were significantly attenuated in *RhoA^cKO^* mice compared with controls. Consistently, levels of the SASP cytokines IL-1β and IL-6 in NALF were markedly lower in HDM-treated *RhoA^cKO^* mice (Figure 5F). To further confirm these findings, we employed our ALI culture system using primary NECs derived from WT mice for in vitro analysis. Following full differentiation, ALI-NECs were stimulated with IL-13 (10 ng/mL) in the presence or absence of the ROCK inhibitor Fasudil (10 μM) [42,43]. IL-13 exposure markedly increased senescence, as evidenced by enhanced SA-β-Gal staining (Figure 5G,H) and elevated expression of p16 (Figure 5G,I), p21 (Figure 5G,J), and γH2AX (Figure 5G,K), whereas Fasudil co-treatment substantially reduced these markers. Moreover, IL-13-induced secretion of SASP cytokines IL-1β and IL-6 into the culture supernatant was significantly attenuated by Fasudil (Figure 5L). Collectively, these findings demonstrate that RhoA/ROCK signaling promotes epithelial senescence and SASP activation both in vitro and in vivo analyses.

### 3.6. Genetic Elimination of Senescent Cells Alleviates Allergic Inflammation, Oxidative Stress, and Epithelial Remodeling

**Figure 6:**
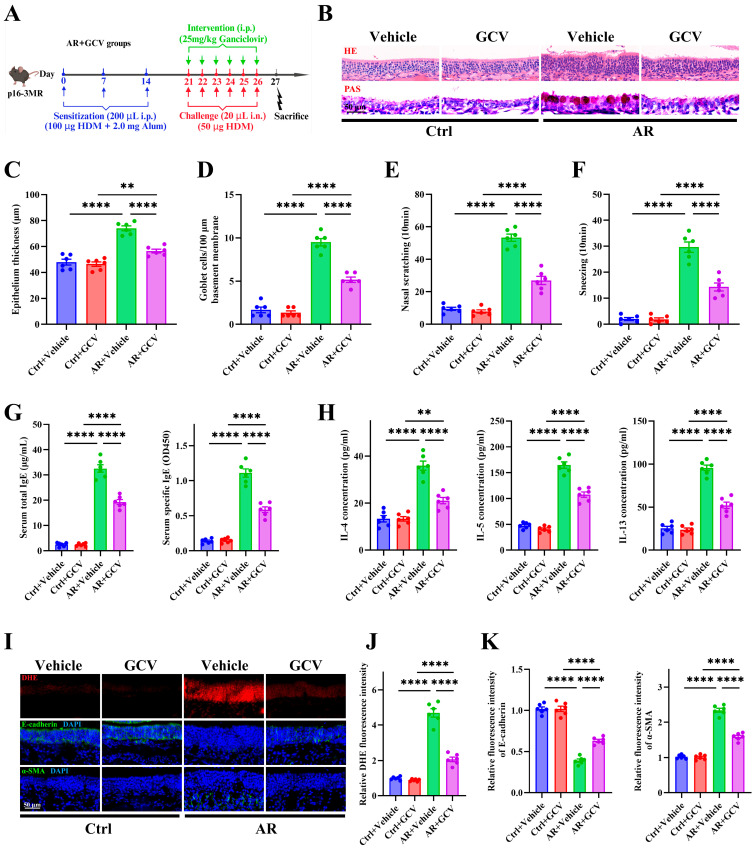
Genetic elimination of senescent cells alleviates allergic inflammation, oxidative stress, and epithelial remodeling. (**A**) Schematic of AR induction and GCV intervention protocol in *p16-3MR* transgenic mice. (**B**) Representative images of H&E and PAS staining in nasal mucosa. (**C**,**D**) Quantification of epithelial thickness (**C**) and goblet cell density (**D**). (**E**,**F**) Quantification of nasal scratching (**E**) and sneezing frequencies (**F**). (**G**) ELISA quantification of serum total and HDM-specific IgE concentrations. (**H**) ELISA quantification of IL-4, IL-5, and IL-13 levels in nasal lavage fluid. (**I**) Representative images of DHE staining and immunofluorescence staining of E-cadherin and α-SMA expression in nasal mucosa. (**J**,**K**) Quantification of DHE (**J**), E-cadherin and α-SMA (**K**) fluorescence intensity. *n* = 6. Data are presented as mean ± SEM. ** *p* < 0.01; **** *p* < 0.0001.

To explore the contribution of cellular senescence to epithelial remodeling and allergic inflammation in AR, we employed *p16-3MR* transgenic mice, which enable visualization and selective ablation of senescent cells through GCV treatment [44] (Figure 6A). Successful elimination of p16^+^ senescent cells in *p16-3MR* mice was confirmed by reduced SA-β-Gal staining (Figure S4A,B). Compared with control littermates, GCV-treated *p16-3MR* mice exhibited markedly attenuated epithelial thickening and goblet cell hyperplasia, as shown by H&E (Figure 6B,C) and PAS staining (Figure 6B,D). These mice also displayed significantly reduced nasal scratching (Figure 6E) and sneezing frequencies (Figure 6F). Consistently, serum total and HDM-specific IgE levels (Figure 6G) and Th2 cytokines (IL-4, IL-5, and IL-13) in NALF (Figure 6H) were significantly decreased following senescent cell ablation. Moreover, GCV-treated *p16-3MR* mice demonstrated reduced epithelial oxidative stress, evidenced by lower ROS levels (Figure 6I,J), and attenuated epithelial remodeling, reflected by restored E-cadherin and decreased α-SMA expression (Figure 6I,K). These findings indicate that epithelial senescence contributes to Th2 inflammation, oxidative stress, and airway remodeling in AR.

### 3.7. PRKN Emerges as a Key Node in RhoA-Regulated Senescence Pathways in AR

**Figure 7:**
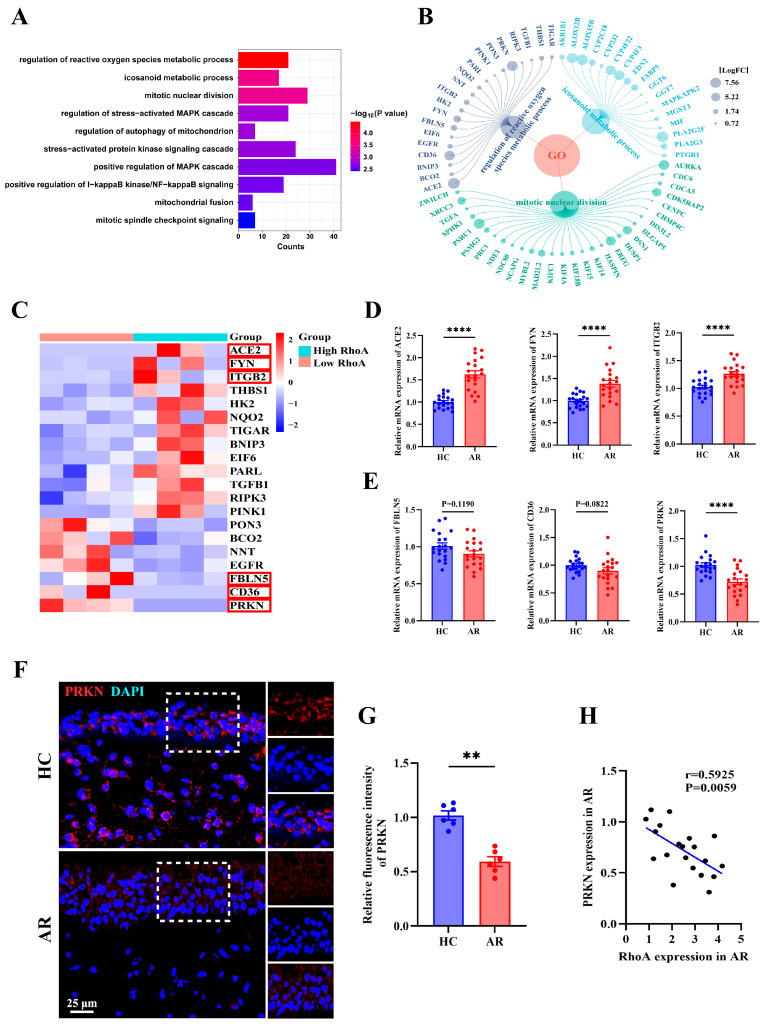
PRKN emerges as a key node in RhoA-regulated senescence pathways in AR. (**A**) Gene Ontology enrichment analysis showing top enriched biological processes associated with cellular senescence in the high-RhoA AR group. (**B**) Visualization of differentially expressed genes enriched in Top3 senescence-associated pathways. (**C**) Heatmap showing the expression of selected oxidative stress-related genes. (**D**,**E**) qRT-PCR analysis of ACE2, FYN, ITGB2 (**D**), FBLN5, CD36, and PRKN (**E**) expression in HC and AR nasal mucosa (*n* = 20). (**F**,**G**) Representative immunofluorescence images (**F**) and quantification (**G**) of PRKN expression in HC and AR nasal mucosa (*n* = 6). (**H**) Correlation analysis between RhoA and PRKN mRNA expression in AR patients (*n* = 20). Data are presented as mean ± SEM. ** *p* < 0.01; **** *p* < 0.0001.

To further elucidate the downstream molecular mechanisms by which RhoA/ROCK signaling regulates epithelial senescence, we performed Gene Ontology (GO) enrichment analysis using the same dataset shown in Figure 4A. Enriched biological processes in AR patients with high RhoA expression included regulation of reactive oxygen species (ROS) metabolism, icosanoid metabolic process, and mitotic nuclear division (Figure 7A). Differentially expressed genes (DEGs) from these top pathways are displayed in Figure 7B. Focusing on the “regulation of reactive oxygen species metabolic process” pathway, we visualized up- and downregulated genes in a heatmap, highlighting ACE2, FYN, ITGB2, FBLN5, CD36, and PRKN (Figure 7C). These key DEGs were further validated by qRT-PCR (Figure 7D,E). Among them, PRKN, a critical regulator of mitophagy and mitochondrial homeostasis, was significantly downregulated in AR nasal mucosa compared with HCs (Figure 7E). Reduced PRKN expression within the nasal epithelium of AR patients was confirmed by immunofluorescence staining (Figure 7F,G). Notably, PRKN mRNA levels negatively correlated with RhoA expression in AR nasal tissues (Figure 7H). Together, these findings suggest that RhoA/ROCK signaling may promote epithelial senescence in AR by suppressing PRKN-mediated ROS and mitochondrial homeostasis.

### 3.8. PRKN Overexpression Alleviates IL-13-Induced Mitochondrial Dysfunction, Oxidative Stress, and Epithelial Senescence in HNEpCs

**Figure 8:**
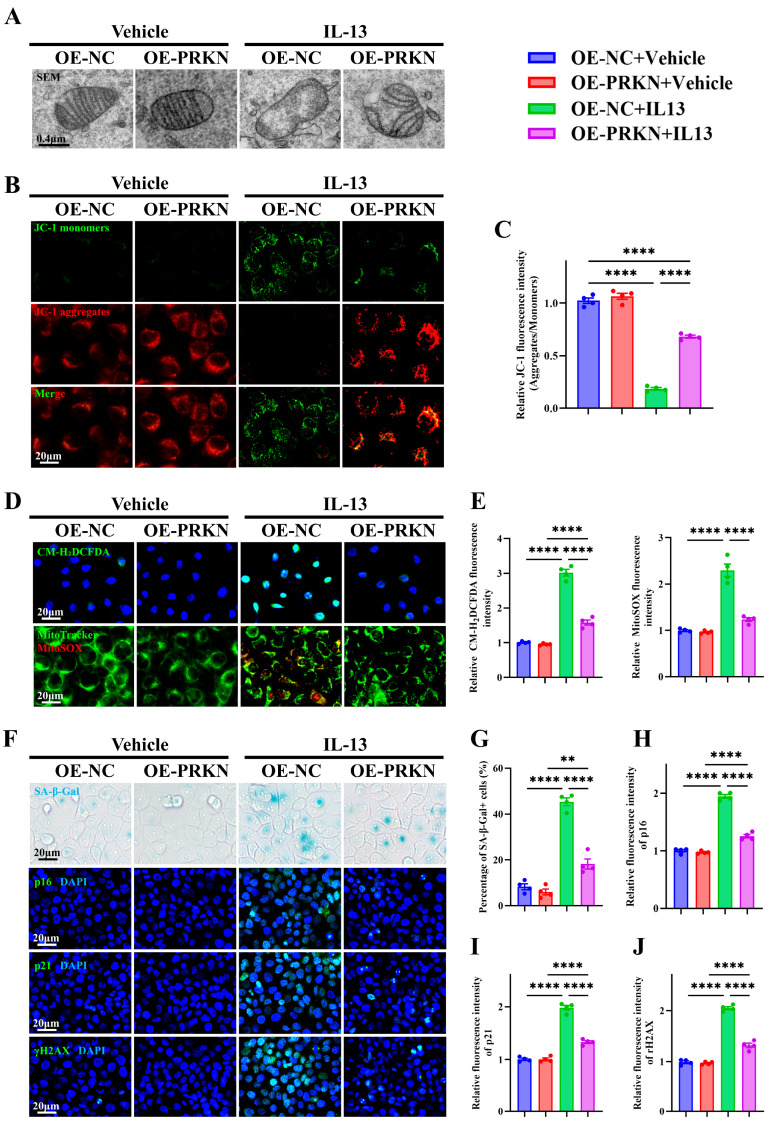
PRKN overexpression alleviates IL-13-induced mitochondrial dysfunction, oxidative stress, and epithelial senescence in HNEpCs. (**A**) Representative images of transmission electron microscopy in PRKN-overexpressing and control HNEpC. (**B**,**C**) Representative images (**B**) and quantification (**C**) of JC-1 staining. (**D**,**E**) Representative images (**D**) and quantification (**E**) of H_2_DCFDA and MitoTracker/MitoSOX staining. (**F**) Representative images of SA-β-Gal staining and immunofluorescence staining for p16, p21, and γH2AX. (**G**–**J**) Quantification of SA-β-Gal^+^ cells (**G**) and fluorescence intensity of p16 (**H**), p21 (**I**), and γH2AX (**J**). *n* = 4. Data are presented as mean ± SEM. ** *p* < 0.01; **** *p* < 0.0001.

To determine whether PRKN functions as a downstream effector linking RhoA/ROCK signaling to epithelial senescence via ROS regulation, we generated PRKN-overexpressing HNEpCs (OE-PRKN HNEpCs), as confirmed by qRT-PCR and Western blot analysis (Figure S5A,B). TEM revealed that IL-13 stimulation induced pronounced mitochondrial swelling and cristae disruption, hallmarks of mitochondrial damage, which were markedly alleviated in OE-PRKN HNEpCs (Figure 8A). Consistently, IL-13 exposure caused a shift from red JC-1 aggregates to green monomers, indicating a loss of mitochondrial membrane potential, whereas PRKN overexpression restored mitochondrial polarization (Figure 8B,C). Similarly, both total intracellular ROS (CM-H_2_DCFDA) and mitochondrial-specific ROS (MitoSOX) were markedly increased after IL-13 stimulation but significantly reduced in OE-PRKN HNEpCs (Figure 8D,E). SA-β-Gal staining further demonstrated that IL-13 treatment induced a robust accumulation of senescent cells, which was substantially diminished in OE-PRKN HNEpCs (Figure 8F,G). Immunofluorescence staining confirmed that PRKN overexpression abrogated IL-13-induced upregulation of the canonical senescence markers p16, p21, and γH2AX (Figure 8F,H–J). Together, these findings indicate that PRKN mitigates IL-13-induced mitochondrial dysfunction, oxidative stress, and epithelial senescence, linking RhoA/ROCK signaling to mitochondrial homeostasis in AR.

## 4. Discussion

While the role of Th2 immune responses and IgE in AR is well established [3,45,46,47,48,49], increasing evidence suggests that the nasal epithelium itself plays an active role in disease pathogenesis [47,50]. Disruption of epithelial barrier integrity, through tight-junction breakdown, impaired mucociliary clearance, and increased allergen penetration, has been implicated in AR [51,52,53]. Epithelial oxidative stress and mitochondrial dysfunction also contribute to chronic inflammation and remodeling [50,51,54,55]. However, how oxidative injury and epithelial stress responses translate into persistent mucosal remodeling remains poorly defined. These gaps hinder understanding of why some patients develop severe or refractory disease despite conventional therapy. Thus, elucidating the upstream epithelial mechanisms that drive remodeling, oxidative stress, and chronic inflammation is critical for identifying novel therapeutic targets. In this study, we identify RhoA/ROCK signaling as a pivotal upstream regulator of epithelial remodeling in AR, acting through oxidative stress and PRKN-dependent cellular senescence. By integrating human clinical samples, genetically modified mouse models, pharmacological inhibition strategies, and primary NEC cultures, we delineate a previously unrecognized molecular pathway linking Th2-type allergic inflammation with epithelial dysfunction. Specifically, we propose a RhoA/ROCK/PRKN-regulated ROS–senescence cascade as a central driver of pathological epithelial remodeling in AR.

Similar to previous findings, we found that the nasal epithelium in AR undergoes marked remodeling characterized by epithelial thickening, goblet cell hyperplasia, and epithelial–mesenchymal transition (EMT)-like changes, accompanied by elevated ROS accumulation and Th2 cytokine expression [3,47,48,49,50,51,54,55]. Particularly, these findings reveal that oxidative stress and epithelial structural reprogramming are central, interlinked features of AR pathogenesis, highlighting epithelial dysfunction, not just immune activation, as a critical and previously underappreciated driver of chronic allergic inflammation. Importantly, transcriptomic and protein-level analyses identified RhoA/ROCK signaling as one of the most significantly upregulated genes in AR nasal mucosa, with activation localized primarily to the epithelium and correlating with clinical severity. This observation aligned with prior studies in asthma, where RhoA/ROCK activation has been shown to impair epithelial integrity through cytoskeletal rearrangement, eosinophil accumulation and tight junction destabilization [8,9,10,56,57], and provided the first evidence that epithelial RhoA activation is directly linked to allergic inflammation in human AR. The positive correlation between RhoA expression and clinical indices (TNSS, RQLQ) underscored its potential as a biomarker of disease activity.

Genetic and pharmacologic inhibition of RhoA/ROCK signaling revealed its central role in orchestrating epithelial remodeling, oxidative stress, and Th2 inflammation in AR. Epithelial-specific deletion of RhoA or treatment with the ROCK inhibitor Fasudil markedly attenuated nasal inflammation, epithelial thickening, goblet cell hyperplasia, and type 2 cytokine production, underscoring that epithelial RhoA activation is not merely a downstream consequence but an upstream driver of allergic pathology. These results expanded prior and demonstrated its functional involvement in allergic inflammation in vivo [8,14,56]. Of interest, the reduction in ROS accumulation and restoration of epithelial integrity following RhoA/ROCK inhibition highlight the pathway’s link to epithelial oxidative injury and barrier dysfunction. Importantly, the in vitro ALI model confirmed that these effects are epithelial-intrinsic, as Fasudil restored TEER values and reversed IL-13-induced ROS and EMT-like changes. Collectively, these findings establish epithelial RhoA/ROCK activation as a key mechanistic node coupling epithelial remodeling and oxidative stress to Th2-driven inflammation, suggesting that targeted inhibition of this pathway could be a viable therapeutic strategy for allergic airway diseases.

One of the striking findings is the increased cellular senescence in AR and closely regulated by RhoA/ROCK signaling. Cellular senescence is increasingly recognized as a pathogenic mechanism in chronic airway diseases, including asthma, pulmonary fibrosis and COPD, but has been largely overlooked in AR [16,23,58,59]. Senescent epithelial cells exhibit irreversible growth arrest while remaining metabolically active, and secrete SASPs, a broad array of pro-inflammatory and tissue-modifying factors [60,61,62]. Transcriptomic and histological analyses revealed strong enrichment of senescence-associated pathways and accumulation of p16^+^, p21^+^, and γH2AX^+^ epithelial cells in AR nasal mucosa, particularly in patients with high RhoA expression. The positive correlation between RhoA activation and senescence markers, together with elevated SASP cytokines (IL-1β and IL-6), suggests that RhoA-driven epithelial stress contributes to a self-perpetuating inflammatory milieu. Functionally, both in vivo (*RhoA^cKO^* mice) and in vitro (ALI-NECs) experiments confirmed that RhoA/ROCK signaling acts as an upstream regulator of allergen- or IL-13-induced epithelial senescence, as genetic deletion or pharmacologic inhibition markedly reduced senescence and SASP induction. These findings identify epithelial RhoA activation as a mechanistic bridge linking oxidative stress to mitochondrial dysfunction and cellular senescence, thereby promoting chronic inflammation and epithelial remodeling in AR. Importantly, they introduce epithelial senescence as a novel pathogenic process, beyond immune activation, that contributes to persistent Th2 inflammation and epithelial remodeling.

We also provided direct evidence that senescent epithelial cells actively contribute to allergic inflammation, oxidative stress, and epithelial remodeling in AR. Specifically, we used the transgenic senescence reporter mouse line *p16-3MR* and evaluated the role of senescence in AR by removing senescent cells using GCV [63]. We found that selective ablation of senescent cells markedly reduced epithelial thickening, goblet cell hyperplasia, oxidative injury, and Th2 cytokine production following allergen challenge. These results confirm that senescent cells are not passive bystanders but active mediators of chronic inflammation through persistent secretion of SASP factors such as IL-1β and IL-6, which amplify local immune responses and epithelial remodeling [22,44,64]. The observed restoration of epithelial integrity and barrier function after senescent cell clearance underscores the pathogenic role of senescent epithelial populations in sustaining allergic pathology. Together, these findings identify epithelial senescence as a mechanistic driver of epithelial remodeling and Th2 inflammation.

Mechanistically, our transcriptomic and mechanistic analyses identify PRKN as a previously unrecognized node connecting RhoA signaling to epithelial senescence. PRKN is a mitochondrial E3 ubiquitin ligase that facilitates mitophagy and maintains mitochondrial quality control [58,65]. Although extensively studied in neurodegenerative and age-related contexts [66,67,68,69], the role of PRKN in airway epithelial biology has yet to be fully elucidated. We found that PRKN was markedly downregulated in AR nasal mucosa and inversely correlated with RhoA expression. Overexpression of PRKN preserved mitochondrial structure and membrane potential while reducing both total and mitochondrial ROS accumulation, highlighting its role in maintaining mitochondrial quality control through mitophagy. Functionally, PRKN restoration markedly reduced SA-β-Gal activity and expression of p16, p21, and γH2AX, confirming its ability to suppress cytokine-induced senescence. These findings provide direct mechanistic evidence that loss of PRKN contributes to RhoA-driven epithelial senescence in AR and that restoring PRKN activity can reverse mitochondrial and oxidative damage. Collectively, this identifies mitochondrial dysfunction as a pivotal link between epithelial stress and cellular senescence, suggesting that targeting the RhoA-PRKN axis or enhancing mitophagy may offer new therapeutic avenues for allergic airway disease. In addition to PRKN, our transcriptomic analyses identified several ROS-related genes differentially regulated in high-RhoA AR mucosa. Notably, ACE2, a modulator of epithelial oxidative injury and inflammation [70]; FYN, a redox-sensitive tyrosine kinase involved in ROS signaling [71]; and ITGB2, an integrin linked to immune activation and oxidative stress responses [72], may represent additional candidates linking RhoA signaling to epithelial dysfunction. Future studies should explore the functional relevance of those novel targets of RhoA in allergic airway disease.

One conceptual question emerging from our findings is whether increased oxidative stress is a predisposing factor for allergic sensitization, or instead a downstream consequence of ongoing allergic inflammation [20,67]. While our current cross-sectional analyses cannot fully resolve this causality, two plausible and non-mutually exclusive hypotheses can be proposed. First, individuals with inherently lower oxidative stress thresholds—potentially due to genetic or epigenetic predisposition—may experience earlier epithelial barrier breakdown and increased susceptibility to aeroallergen penetration and sensitization [31,73]. Alternatively, once sensitization occurs, Th2 cytokine-driven inflammation may amplify epithelial oxidative damage through RhoA activation, leading to persistent epithelial remodeling and senescence [8,47,60]. Clarifying this sequence of events is important, as it could inform early diagnostic markers and support tailored antioxidant-based interventions in high-risk individuals. Longitudinal studies in sensitization-prone cohorts and oxidative stress-stratified AR subtypes will be essential to address this issue.

Several limitations should be acknowledged. Although epithelial-specific RhoA deletion demonstrates causality, the contribution of RhoA signaling in other structural and immune cells remains to be investigated. The modest sample size in human transcriptomic analyses may limit statistical power, and the correlation between RhoA and senescence markers warrants confirmation in larger and more diverse cohorts. Moreover, pharmacologic inhibition with fasudil, while supportive, may exert off-target effects. In addition, cytokine levels in nasal secretions were not normalized to total protein or IgA content, which may have affected absolute concentration comparisons due to variable mucus volume. Finally, this study focused primarily on Th2-mediated inflammation; future work should examine whether RhoA-driven senescence also influences non-Th2 inflammatory pathways and steroid responsiveness. Looking forward, future studies should explore how RhoA/ROCK signaling transcriptionally represses PRKN and whether epigenetic or post-translational mechanisms are involved. Integration of single-cell and spatial transcriptomic approaches will help delineate epithelial subpopulations prone to senescence and their spatial relationship to immune infiltrates. Translationally, preclinical evaluation of ROCK inhibitors or mitophagy-enhancing agents may offer new therapeutic strategies for patients with refractory or steroid-insensitive AR.

In summary, this work establishes a mechanistic framework in which RhoA/ROCK-driven epithelial senescence links oxidative stress to Th2 inflammation and tissue remodeling in AR and identifies PRKN as a novel mitochondrial checkpoint with therapeutic potential to restore epithelial homeostasis and attenuate chronic allergic inflammation.

## Data Availability

The datasets generated during and/or analyzed during the current study are available from the corresponding author on reasonable request. All RNA-sequence original data have been mapped to the CNCB Sequence Read Archive (BioProject ID: PRJCA038464 for human samples).

## Supplementary Materials

The following supporting information can be downloaded at: https://www.mdpi.com/article/10.3390/antiox15010077/s1.

## Abbreviations

AR	Allergic rhinitis
HC	Healthy control
ALI	Air–liquid interface
CC10	Club cell secretory protein 10
EMT	Epithelial–mesenchymal transition
GCV	Ganciclovir
H&E	Hematoxylin and eosin
HDM	House dust mite
HNEpCs	Human nasal epithelial cells
NALF	Nasal lavage fluid
NECs	Nasal epithelial cells
RhoA	Ras homolog family member A
ROCK	Rho-associated coiled-coil-containing protein kinase
ROS	Reactive oxygen species
RQLQ	Rhinoconjunctivitis quality of life questionnaire
SASP	Senescence-associated secretory phenotype
SEM	Standard error of the mean
TEER	Transepithelial electrical resistance
TEM	Transmission electron microscopy
TNSS	Total nasal symptom score
qRT-PCR	Quantitative reverse transcription PCR
COPD	Chronic obstructive pulmonary disease
WT	Wild-type
DEGs	Differentially expressed genes
GO	Gene Ontology
