# Supplementary material for: Oxidative Stress and PRKN-Mediated Senescence Link RhoA/ROCK Signaling to Epithelial Remodeling in Allergic Rhinitis
Source: Antioxidants (Basel). 2026 Jan 7;15(1):77. doi: 10.3390/antiox15010077 (PMC12837145; doi:10.3390/antiox15010077)

Online Repository

Oxidative Stress and PRKN-Mediated Senescence Link RhoA/ROCK Signaling to Epithelial Remodeling in Allergic Rhinitis

Xuan Yuan, MD[a, b], Wei Zhong, MD[b], Shaobing Xie, MD, PhD[a, b], Liyuan Liu, BS[a], Wenjing Gu, MD, PhD[a, c], Yixiang Zeng, BS[a], Hua Zhang, MD, PhD[b, d], Weihong Jiang, MD, PhD[b, d], Zhihai Xie, MD, PhD[b, d#], Peisong Gao, MD, PhD[a#]

[a]Division of Allergy and Clinical Immunology, Johns Hopkins University School of Medicine, Baltimore, MD, 21224, USA.

[b]Department of Otolaryngology Head and Neck Surgery, Xiangya Hospital of Central South University, Changsha, Hunan, 410008, China.

[c]Department of Respiratory Medicine, Children's Hospital of Soochow University, Suzhou, Jiangsu, 215000, China.

[d]Hunan Province Key Laboratory of Otolaryngology Critical Diseases, Xiangya Hospital of Central South University, Changsha, Hunan, 410008, China.

[#]To whom correspondence should be addressed:

Peisong Gao, MD, PhD: The Johns Hopkins Asthma & Allergy Center, 5501 Hopkins Bayview Circle, Room 3B.71, Baltimore, MD 21224, USA. Telephone: 410-550-2124, Email: pgao1@jhmi.edu.

Zhihai Xie, MD, PhD: Department of Otolaryngology Head and Neck Surgery, Xiangya Hospital of Central South University, 87 Xiangya Road, Changsha, Hunan, 410008, China. Email: xiedoctor@csu.edu.cn.

Table S1. Baseline characteristics of study subjects

Variable	HC (n=20)	AR (n=20)	P value
Age, mean (SD), years	35.4 (8.8)	35.3 (8.2)	0.971
Male, No. (%)	10 (50.0)	10 (50.0)	1.000
BMI, mean (SD), kg/m^2	23.5 (3.5)	23.6 (3.3)	0.926
Smoking, No. (%)	0 (0.0)	0 (0.0)	1.000
Asthma, No. (%)	0 (0.0)	0 (0.0)	1.000
AR duration, mean (SD), years	-	9.2 (4.2)	
TNSS score, mean (SD)	-	9.1 (2.3)	
RQLQ score, mean (SD)	-	102.8 (30.4)	

Table S2. Primers used in this study

Gene	Species	Sequence, 5′→3′
RhoA	*Homo sapiens*	Forward: TCGAGGTGGATGGAAAGCAG<br>Reverse: GGCACGTTGGGACAGAAATG
ACE-2	*Homo sapiens*	Forward: TGGGTCTTCAGTGCTCTCTCAG<br>Reverse: CTTGCCGACCTCAGATCTCC
FYN	*Homo sapiens*	Forward: GGACTCACCGTCTTTGGAGG<br>Reverse: AAGGTCCCCGTATGAGACGA
ITGB2	*Homo sapiens*	Forward: GATGACGGCTTCCATTTCGC<br>Reverse: TGGGGATGATCTCGGTGAGT
FBLN5	*Homo sapiens*	Forward: GGAATAAAACACCCGCGAGC<br>Reverse: ACTGGCGATCCAGGTCAAAG
CD36	*Homo sapiens*	Forward: CGCTGAGGACAACACAGTCT<br>Reverse: CTGCCACAGCCAGATTGAGA
PRKN	*Homo sapiens*	Forward: TTGCGTGTGATTTTCGCAGG<br>Reverse: TTTCTCCACGGTCTCTGCAC
p16	*Homo sapiens*	Forward: CTGCCCAACGCACCGAATAG<br>Reverse: TCATCATGACCTGGATCGGC
p21	*Homo sapiens*	Forward: CCTGCCCAAGCTCTACCTTC<br>Reverse: TGGTAGAAATCTGTCATGCTGGT
GAPDH	*Homo sapiens*	Forward: CTCCTCCTGTTCGACAGTCAGC<br>Reverse: CCCAATACGACCAAATCCGTT
α-SMA	*Mus musculus*	Forward: CCCAACTGGGACCACATGG<br>Reverse: TACATGCGGGGGACATTGAAG
E-cadherin	*Mus musculus*	Forward: AAAAGAAGGCTGTCCTTGGC<br>Reverse: GAGGTCTACACCTTCCCGGT
β-actin	*Mus musculus*	Forward: CTATTGGCAACGAGCGGTTC<br>Reverse: AATGCCTGGGTACATGGTGG

Table S3. The antibodies used in this study

Antibody	Dilution	Company
RhoA-GTPase	1:100	New East Biosciences
PRKN	1:100	Cell Signaling Technology
α-SMA	1:100	Proteintech
E-cadherin	1:100	Cell Signaling Technology
γH2AX	1:100	Cell Signaling Technology
p16	1:100	Proteintech
p21	1:100	Invitrogen

FIGURE LEGENDS

Figure S1. Generation and genotyping of *RhoA*^{cKO} mice. (**A**) Schematic diagram depicts the generation of *RhoA*^{cKO} mice by crossing *RhoA*$^{f/f}$ mice with *CC10-CreER*^{TM} mice. (**B**) Specific depletion of RhoA^{+} cells in Club cells were confirmed by genotyping.

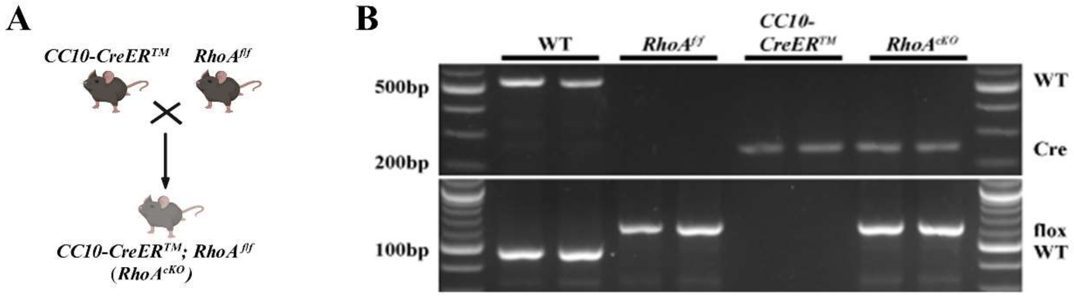

Figure S2. Pharmacological inhibition of RhoA/ROCK signaling ameliorates nasal Th2 inflammation, oxidative stress, and epithelial remodeling in AR. (**A**) Schematic of Fasudil treatment protocol in HDM-induced AR model. (**B**) Representative images of H&E and PAS staining in nasal mucosa. (**C-D**) Quantification of epithelial thickness (**C**) and goblet cell density (**D**). (**E-F**) Quantification of nasal scratching (**E**) and sneezing frequencies (**F**). (**G**) ELISA quantification of serum total and HDM-specific IgE concentrations. (**H**) ELISA quantification of IL-4, IL-5, and IL-13 levels in nasal lavage fluid. (**I**) Representative images of DHE staining and immunofluorescence staining of E-cadherin and α-SMA expression in nasal mucosa. (**J-K**) Quantification of DHE (**J**), E-cadherin and α-SMA (**K**) fluorescence intensity. n=6. Data are presented as mean ± SEM. ****$P < 0.0001$.

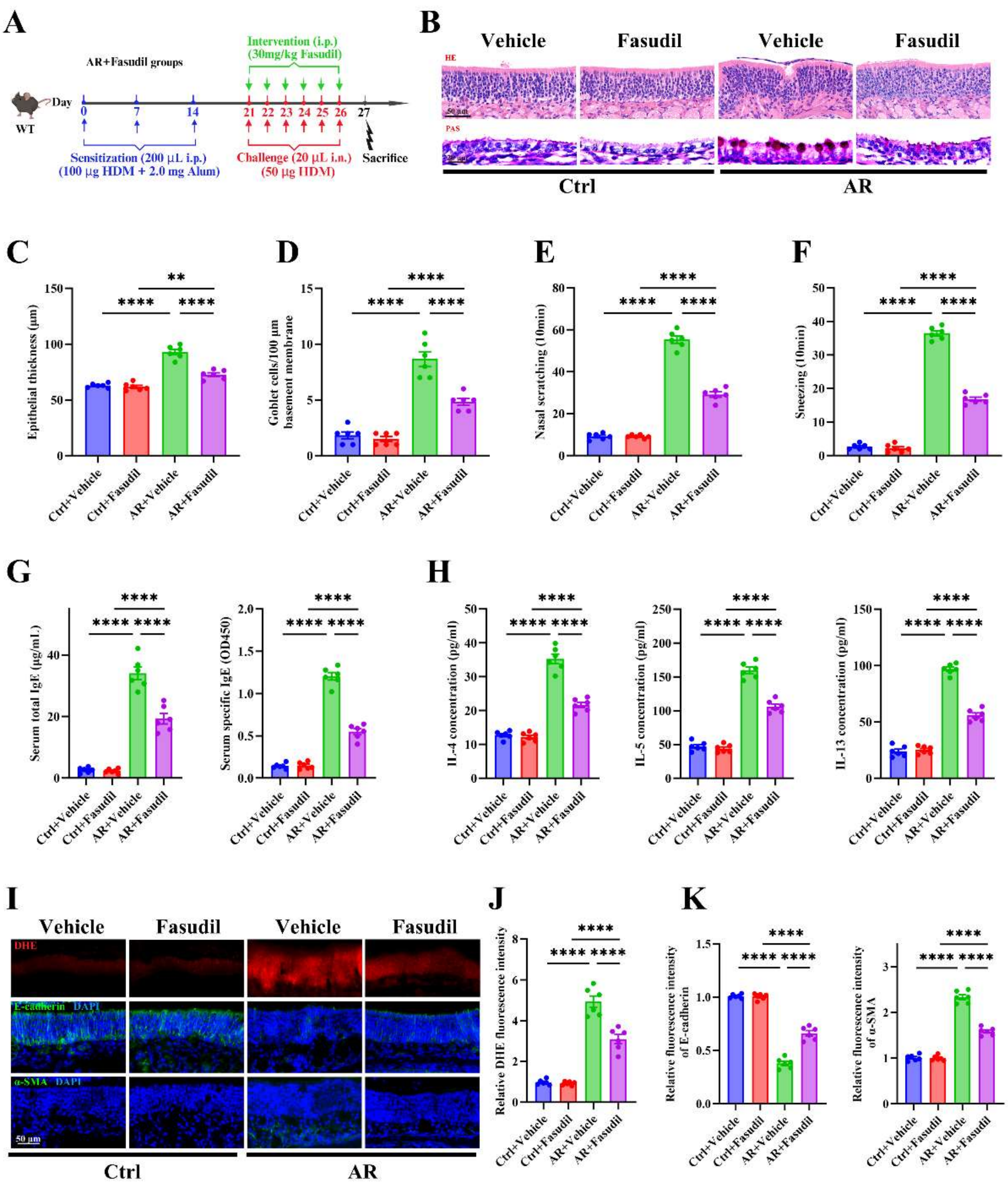

Figure S3. RhoA/ROCK inhibition protects against IL-13–induced ROS and epithelial remodeling in ALI-NECs. (**A**) Schematic of ALI-cultured mouse NEC differentiation and treatment protocol. (**B**) Representative images of CM-H_2DCFDA staining and immunofluorescence staining for E-cadherin and α-SMA expression. (**C-E**) Quantification of fluorescence intensity of CM-H_2DCFDA (**C**), E-cadherin (**D**) and α-SMA (**E**) (n=4). (**F**) Measurement of TEER over time in ALI-NECs (n=6). Data are presented as mean ± SEM; ****P <0 .001; ****P < 0.0001*.

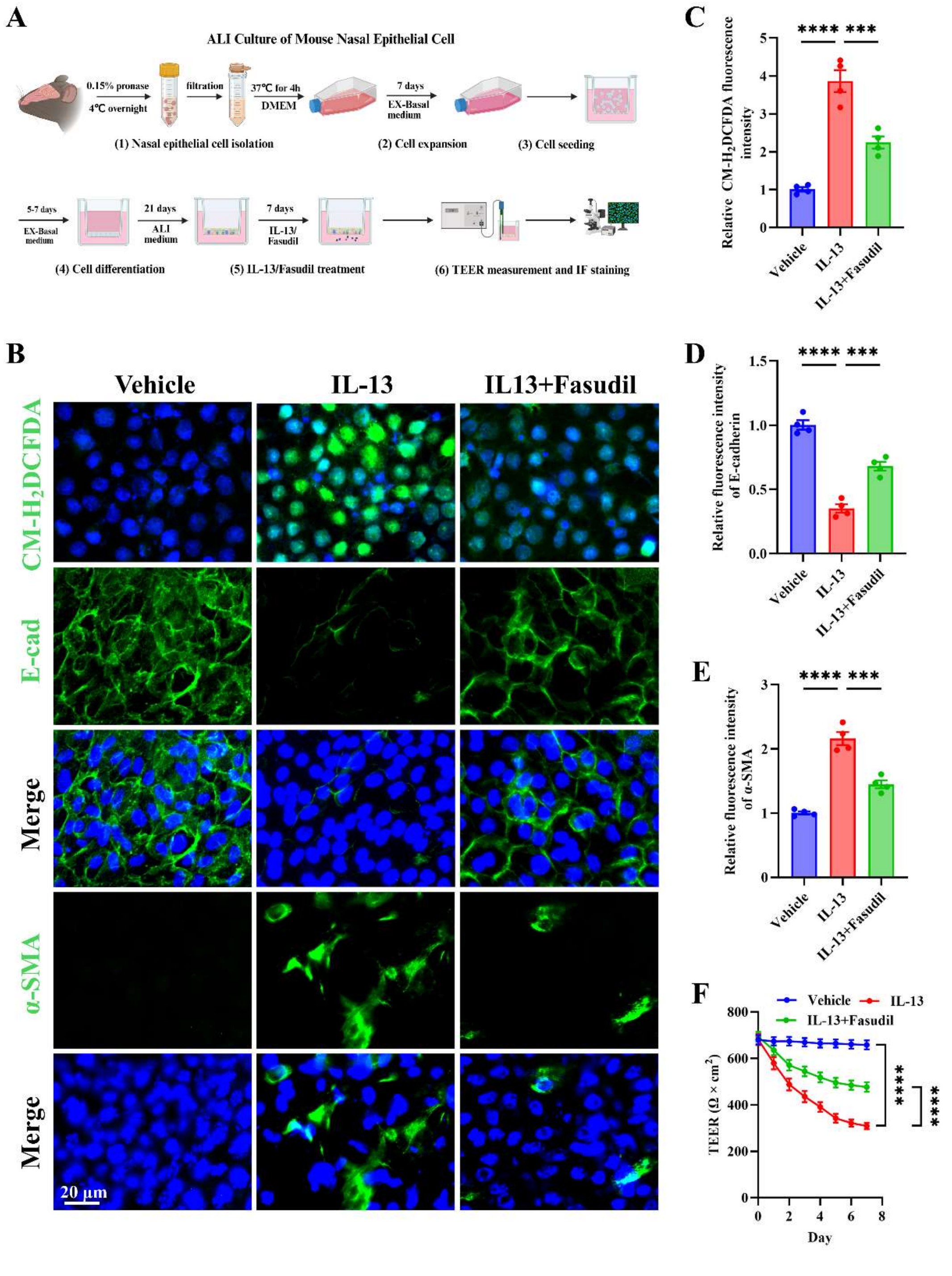

Figure S4. Validation of the successful elimination of p16+ senescent cells in *p16-3MR* mice. (**A-B**) Representative images (**A**) and quantification (**B**) of SA-β-Gal staining in nasal mucosa (n=6). Data are presented as mean ± SEM; $**P<0.01$; $****P<0.0001$.

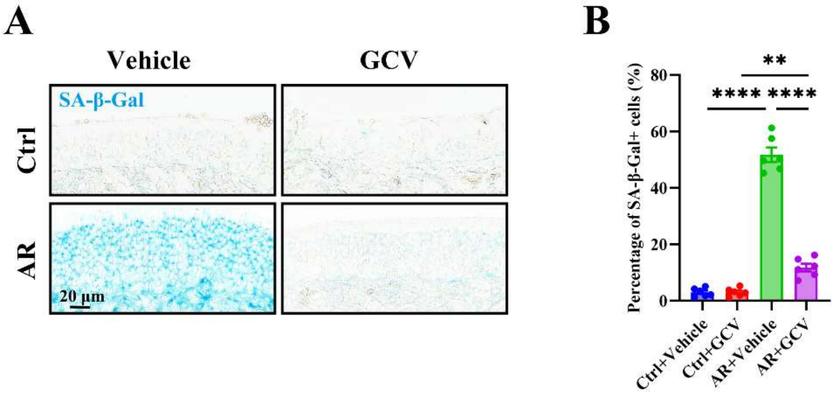

Figure S5. Validation of PRKN overexpression in HNEpCs. (**A**) qRT-PCR analysis of PRKN mRNA expression in OE-NC and OE-PRKN HNEpCs (n=4). (**B**) Representative Western blot images of PRKN protein levels in OE-NC and OE-PRKN groups. Data are presented as mean ± SEM; $**P<0.01$.

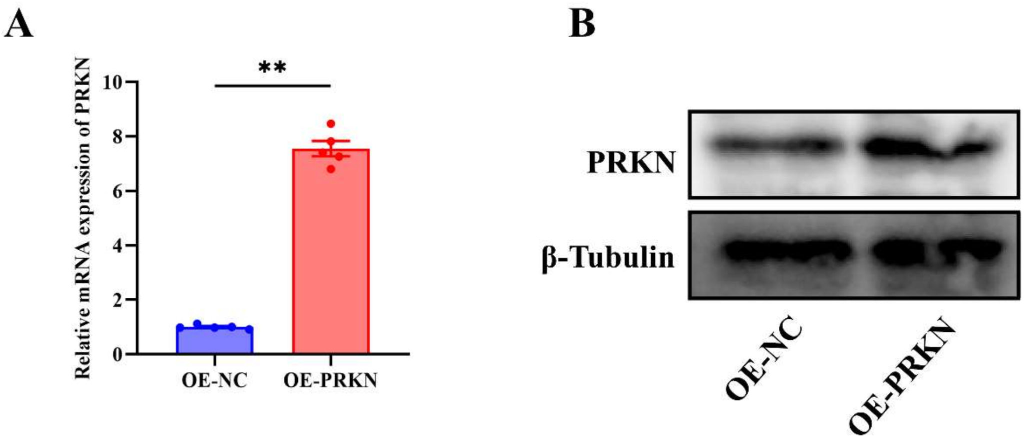

Supplement: Supplementary file 1 [file antioxidants-15-00077-s001.zip › antioxidants-4028451-supplementary.pdf]
